# Secondary lung abscess caused by an esophagorespiratory fistula in a patient with a past history of heavy alcohol consumption

**DOI:** 10.1002/jgf2.70056

**Published:** 2025-08-23

**Authors:** Shuhei Nozaki, Taku Yabuki, Taro Shimizu

**Affiliations:** ^1^ Internal Medicine Katori City Tonosho Town Hospital Association Chiba Japan; ^2^ Internal Medicine National Hospital Organisation Tochigi Medical Center Tochigi Japan; ^3^ Department of Diagnostic and Generalist Medicine Dokkyo Medical University Tochigi Japan

**Keywords:** esophageal cancer, esophagorespiratory fistula, heavy alcoholic drinker, secondary lung abscess

## Abstract

A man in his 60s was ultimately diagnosed with a secondary lung abscess caused by an esophagorespiratory fistula. On admission, however, he had initially been diagnosed with a primary lung abscess because of aspiration, given his history of alcohol use. Secondary lung abscesses can result from various underlying conditions. Among these, esophagorespiratory fistulas are significant causes and are often associated with esophageal cancers. An intriguing aspect of this case is that secondary lung abscesses originating from esophageal cancer can occur under similar conditions as aspiration‐related pulmonary suppuration. Given the difficulty in distinguishing between the two based solely on imaging, physicians should exercise caution when encountering patients with a history of heavy alcohol consumption.

## Graphical abstract

This case highlights the importance of considering malignancy‐related esophagorespiratory fistulas in patients with presumed aspiration‐related lung abscesses and heavy alcohol use.

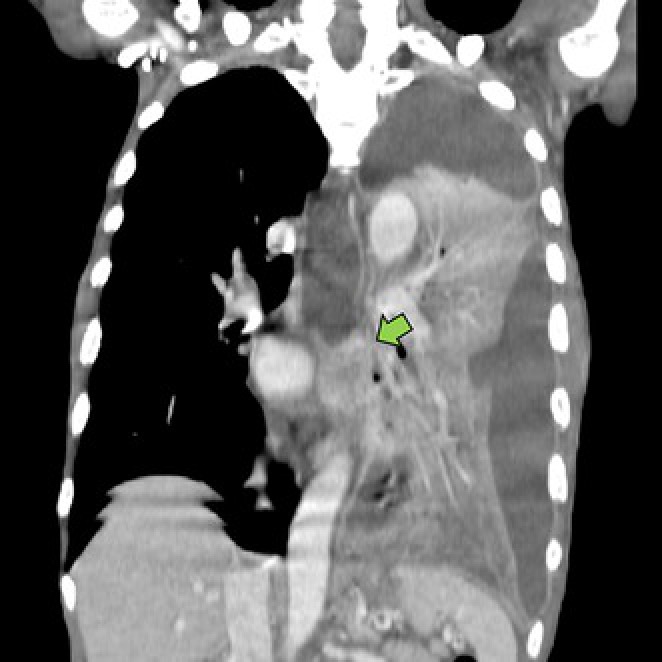

## 1 BACKGROUND

Lung abscesses are a form of tissue liquefaction in the lung, characterized by cavities containing necrotic debris or fluid caused by microbial infection. These abscesses often result from bacterial infection that creates a cavity within the lung. Lung abscesses are classified as primary or secondary. Primary lung abscesses, typically caused by aspiration, account for 60% of all lung abscesses and are associated with advanced age or alcoholism.^1^, ^2^ Secondary lung abscesses, accounting for the remaining 40%, may arise from direct overflow from esophagorespiratory fistulas or subdiaphragmatic abscesses, preexisting lung pathologies such as bronchiectasis, hematogenous spread including infective endocarditis, and obstructive pneumonia because of tumors.^1^, ^2^ Careful differentiation between primary and secondary lung abscesses is essential; however, distinguishing the two can sometimes be challenging, leading to potential misdiagnosis. We herein report a case of a secondary lung abscess because of an esophagorespiratory fistula, where distinguishing it from a primary lung abscess proved difficult.

## 2 CASE PRESENTATION

A man in his 60s with alcohol dependency presented with tachypnea. His history included alcoholic liver disease, epilepsy, and heavy smoking (40 cigarettes/day from ages 20 to 57). He consumed more than 40 g of alcohol per day—Japanese sake—from the age of 20 until a few years ago, but had reportedly reduced his intake in recent years. During a routine visit, he appeared tachypneic without fever, cough, or sputum. The patient's history revealed no dysphagia and no coughing after swallowing. Vital signs showed a pulse of 104 beats/min, blood pressure of 104/78 mmHg, respiratory rate of 28 breaths/min, and SpO2 of 91% on room air. Physical examination revealed diminished breath sounds over the entire left lung; the oral cavity was somewhat unhygienic with prominent gingivitis, and although the dentition was largely intact, several teeth showed dental caries.

**FIGURE 1:**
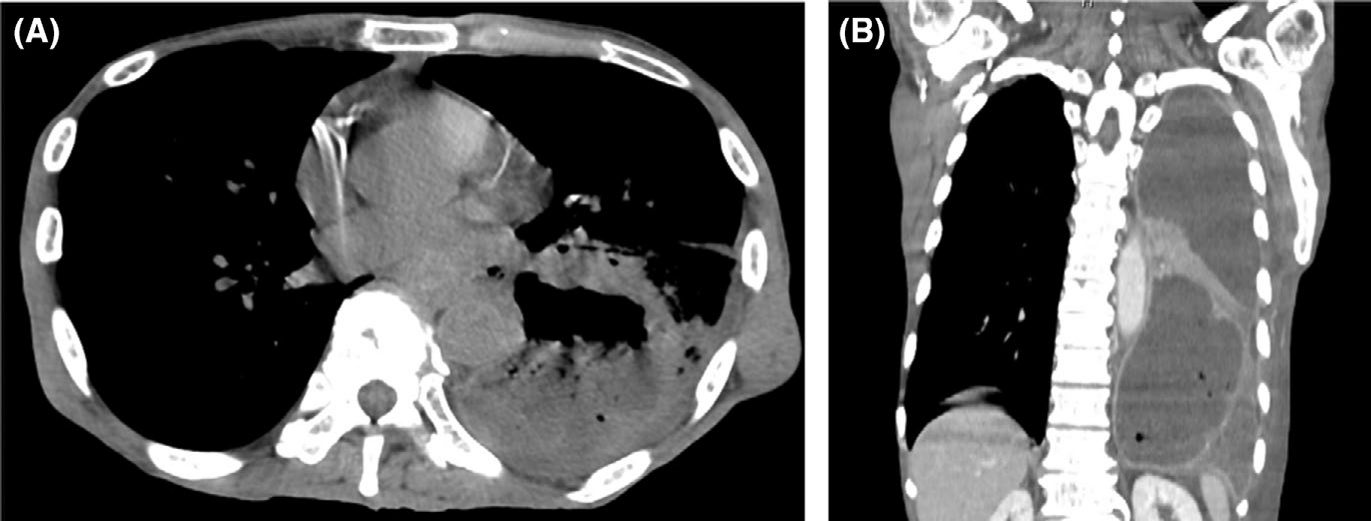
Non‐contrast chest CT images (A) obtained at admission revealed a large lung abscess in the left lower lobe accompanied by pneumothorax. Subsequent contrast‐enhanced chest CT images (B) provided clearer delineation of the abscess and demonstrated its rupture, resulting in empyema.

Laboratory tests demonstrated leukocytosis with a white blood cell count of 20,200/μL, an elevated C‐reactive protein level of 27.12 mg/dL, and hypoalbuminemia with a serum albumin of 2.5 g/dL. Initial non‐contrast CT revealed a 90‐mm left lower lobe lung abscess accompanied by pneumothorax, prompting hospital admission and antimicrobial therapy (Figure 1A). The abscess was initially presumed to be because of aspiration given his alcohol use history.

**FIGURE 2:**
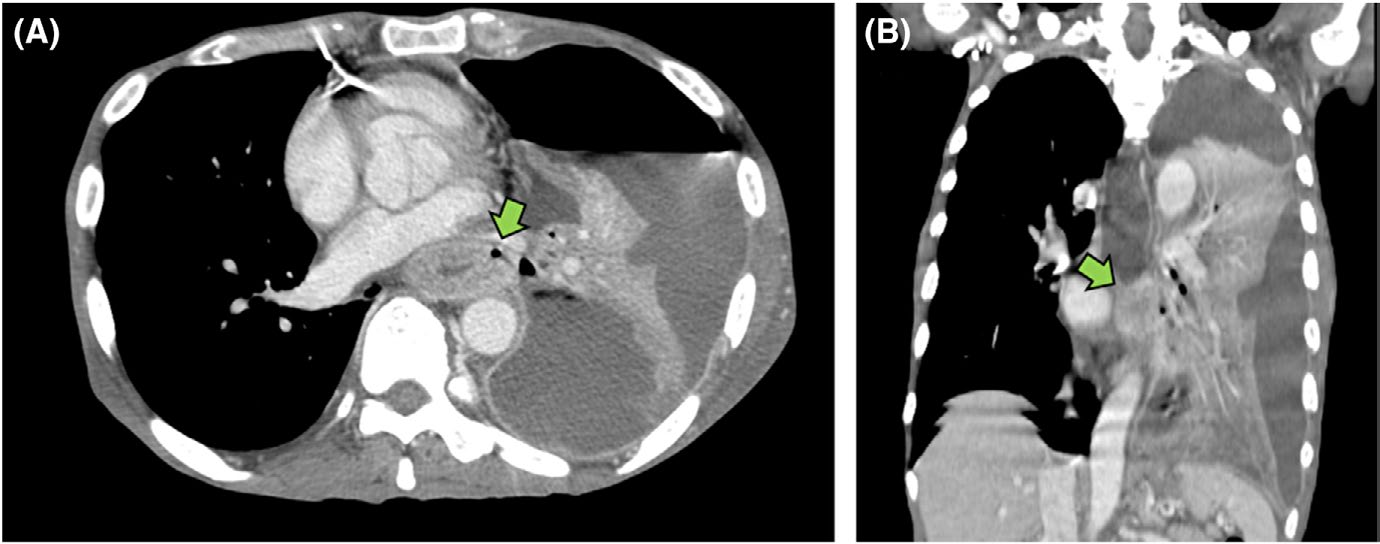
The same contrast‐enhanced CT images revealed circumferential thickening of the esophageal wall and intramural free air (A and B, arrow), suggesting esophageal cancer complicated by esophagorespiratory fistula.

Two days later, contrast‐enhanced CT was performed, revealing extensive pleural effusion, suggesting rupture into the thoracic cavity and empyema (Figure 1B). Worsening respiratory condition with increased oxygen demand necessitated thoracic drainage. A large amount of purulent effusion was evacuated, which improved symptoms. Pleural fluid cultures grew *Enterobacter cloacae*, *Candida albicans*, and *Bacillus subtilis*, requiring escalation of antimicrobial therapy. The enhanced CT also showed esophageal wall thickening and intramural air, raising suspicion of an esophageal fistula because of cancer (Figure 2A,B). After respiratory stabilization, an endoscopy revealed esophageal stricture and irregularities, strongly suggesting esophageal cancer. Because of the high risk of esophageal perforation through friable tumor tissue or exacerbation of a pre‐existing fistula, biopsy was deferred. The lung abscess was confirmed secondary to an esophagorespiratory fistula attributed to esophageal cancer. An esophageal stent was placed, and antimicrobial therapy and drainage were continued. Finally, antimicrobial therapy was mainly based on piperacillin–tazobactam and micafungin.

Further intervention for suspected cancer was considered but declined per the patient's wishes because of his poor condition. Supportive care was prioritized. After 1 month, he transitioned to oral antimicrobials and was discharged. Over the year, esophageal tumor progression caused cachexia and respiratory failure, leading to his death.

## 3 DISCUSSION

Esophagorespiratory fistulas, encompassing tracheoesophageal, bronchoesophageal, and pulmonary‐esophageal fistulas, are often associated with malignancies, with 5%–10% of esophageal cancers presenting with such complications.^3^, ^4^ These fistulas commonly result in lower respiratory tract infections, such as bronchitis and pneumonia.^4^ Although the exact frequency of esophagorespiratory fistulas causing lung abscesses is unclear, several reports, including our case, emphasize their role in severe lung abscess formation, highlighting their significance as a secondary cause of lung abscesses.^5^, ^6^

In this case, on admission, an aspiration‐related primary lung abscess was diagnosed. However, subsequent CT imaging revealed findings suggestive of an esophagorespiratory fistula and esophageal cancer, clarifying the origin of the lung abscess and prompting additional management for the underlying malignancy.

This case underscores the need to consider secondary causes of lung abscesses, particularly in patients with a history of heavy alcohol consumption. We should also remember that heavy alcohol use is a risk factor not only for aspiration‐related lung abscesses but for esophageal cancer itself.^7^ The absence of typical symptoms of esophagorespiratory fistula or esophageal cancer, such as dysphagia, vomiting, and chest pain, contributed to the diagnostic challenge. Furthermore, comprehensive CT evaluation is crucial, as initial assessments may overlook critical findings. In this case, the radiologist's detailed interpretation, which identified the esophagorespiratory fistula and esophageal cancer, was pivotal in achieving the correct diagnosis.

While aspiration is a common cause of lung abscesses, secondary causes such as esophageal cancers and esophagorespiratory fistulas should be carefully considered, particularly in patients with heavy alcohol use. Identifying these specific causes is crucial for appropriately managing both the abscess and the underlying condition.

## 4 CONCLUSION

The significant prevalence of secondary lung abscesses (reported in up to 40% of cases) warrants careful diagnostic consideration.^1^, ^2^ Physicians should evaluate for secondary lung abscesses in all cases of presumed lung abscess. Particularly in heavy alcohol drinkers, there is a heightened risk of esophagorespiratory fistulas because of esophageal cancer, warranting close attention.

## AUTHOR CONTRIBUTIONS

**Shuhei Nozaki:** Conceptualization; writing – original draft; methodology; validation; investigation; visualization; project administration; writing – review and editing. **Taku Yabuki:** Supervision; writing – review and editing. **Taro Shimizu:** Writing – review and editing; supervision.

## CONFLICT OF INTEREST STATEMENT

The authors declare no conflict of interest.

## ETHICS STATEMENT

Ethics approval statement: None.

Patient consent statement: None.

Clinical trial registration: None.

## CONSENT

Written informed consent for publication of this case report and accompanying images was obtained from the patient.

## References

[1] Kuhajda I , Zarogoulidis K , Tsirgogianni K , Tsavlis D , Kioumis I , Kosmidis C , et al. Lung abscess‐etiology, diagnostic and treatment options. Ann Transl Med. 2015;3(13):183. 26366400 10.3978/j.issn.2305-5839.2015.07.08 PMC4543327

[2] Sabbula BR , Rammohan G , Athavale A , Akella J . Lung abscess. StatPearls. Treasure Island (FL): StatPearls Publishing; 2023. https://www.ncbi.nlm.nih.gov/books/NBK547706/ 32310380

[3] Chen YH , Li SH , Chiu YC , Lu HI , Huang CH , Rau KM , et al. Comparative study of esophageal stent and feeding gastrostomy/jejunostomy for tracheoesophageal fistula caused by esophageal squamous cell carcinoma. PLoS One. 2012;7(8):e42766. 22912737 10.1371/journal.pone.0042766 PMC3418295

[4] Balazs A , Kupcsulik PK , Galambos Z . Esophagorespiratory fistulas of tumorous origin. Non‐operative management of 264 cases in a 20‐year period. Eur J Cardiothorac Surg. 2008;34(5):1103–1107. 18678504 10.1016/j.ejcts.2008.06.025

[5] Ghosh S , Handa N , Tale S , Bhalla A . A rare case of lung abscess due to esophageo‐pulmonary fistula in asymptomatic carcinoma of esophagus. Monaldi Arch Chest Dis. 2021;91(4). 10.4081/monaldi.2021.1815 34006040

[6] Keinath K , Porambo M , Nguyen B . Pulmonary abscess secondary to oesophageal carcinoma erosion. BMJ Case Rep. 2020;13(10):e239223. 10.1136/bcr-2020-239223 PMC7604809 33127714

[7] Oze I , Matsuo K , Wakai K , Nagata C , Mizoue T , Tanaka K , et al. Alcohol drinking and esophageal cancer risk: an evaluation based on a systematic review of epidemiologic evidence among the Japanese population. Jpn J Clin Oncol. 2011;41(5):677–692. 21430021 10.1093/jjco/hyr026

